# “Hey, that could be me”: The role of similarity in narrative persuasion

**DOI:** 10.1371/journal.pone.0215359

**Published:** 2019-04-18

**Authors:** Joëlle Ooms, John Hoeks, Carel Jansen

**Affiliations:** 1 University of Groningen, Groningen, The Netherlands; 2 Language Centre, Stellenbosch University, Stellenbosch, South Africa; University of Göttingen, GERMANY

## Abstract

Stories are often used in health communication because of accumulating evidence of their potential to affect people’s attitudes and health behavioral intentions. Similarity between the reader and the story’s protagonist appears to positively influence narrative persuasion, but the exact role of similarity on persuasive outcomes is debated, as some research finds clear effects of similarity manipulations whereas others do not. Possibly, these mixed results were found because the similarity manipulations were not always relevant to the topic of the story. We conducted an experiment (N = 582) in which we varied the age and gender of the protagonist, features that were of central relevance to the story’s topic, namely breast cancer versus testicular cancer. There were two groups of participants: 324 students (mean age: 21.46 years) and 258 older adults (mean age: 56.83 years). Age similarity (but not gender similarity) had an effect on identification with the protagonist, transportation (i.e. the experience of being absorbed into a story), and the intention to donate, but only for students. For older adults, age or gender of the protagonist did not seem to matter, as nearly no differences in persuasive measures were found. As far as the underlying mechanism is concerned, the results of structural equation modeling showed that the concept of ‘perceived similarity’ would be a relevant addition to models of narrative persuasion, as it was significantly related to the narrative processes of transportation and identification, which, in turn, predicted attitudes and behavioral intentions, both directly—in the case of transportation—or indirectly, via the emotion of compassion. We conclude that both manipulated and perceived similarity are important for narrative persuasion, and that it should be kept on the research agenda of health communication.

## Introduction

Stories, or narratives, are increasingly studied in a health context by scholars from different disciplines [1, 2]. A well-known type of narrative in this domain is the *illness narrative—*also referred to as health narrative, testimonial, anecdote or patients story—which can be defined as a “first-person story about experiences with illness and its personal consequences” ([3]: p 28). Illness narratives can thus be seen as a form of meaning making which enables patients to give voice to their suffering [4, 5]. However, these narratives can also help other people in the decision making process concerning their own health.

Empirical research in persuasive health communication has shown that narratives have the potential to affect people’s attitudes and health behavioral intentions [6, 7]. Such effects may be achieved by being *transported* into the narrative [8], and by *identification* with the protagonist [9]. Transportation is defined as “a convergent process, where all mental systems and capacities become focused on events occurring in the narrative” ([8]: p 701). Identification is “a process that consists of increasing loss of self-awareness and its temporary replacement with heightened emotional and cognitive connections with a character” ([9]: p 251). Given the key roles of these two concepts in narrative persuasion, it is of great interest to know how transportation and identification can be maximized. It has been suggested that the extent to which the protagonist in a narrative is perceived as *similar* to the readers can positively influence both identification and transportation [10]. Though similarity is sometimes seen as an integral part of identification [11], it has been convincingly argued that they are conceptually and empirically distinct constructs [12, 13]: Identification is a process whereby a viewer or reader experiences the narrative from the inside, while perceived similarity involves the viewer or reader acting as an external observer to the narrative, thus from the outside [9, 12, 13].

Empirical evidence for the effects of *similarity manipulations* on transportation, identification, and, subsequently, persuasion is mixed, however [2]. Nevertheless, as several studies show a positive correlation between perceived similarity and identification [13–15], Chen et al. [15] conclude that “the need to develop other strategies for fostering *similarity perceptions* remains a high research priority” (p. 710). The current paper answers this call by investigating the effects of manipulated similarity on perceived similarity, identification and transportation, and on persuasive outcomes of narratives. Moreover, we look into the mechanisms behind the potential persuasive impact of perceived similarity.

### Manipulated similarity and perceived similarity

The role of similarity in narrative persuasion has received substantial attention in the literature. De Graaf and colleagues [2] performed a systematic review on studies that compared a narrative with a similar protagonist to a narrative with a dissimilar protagonist. They found that similarity manipulations hardly ever affected identification, transportation, or persuasion (i.e. beliefs, attitudes, intentions or actual behaviors). Results of a recent study [16] are in line with these conclusions, finding no effects for four different manipulations of similarity on identification or on measures of persuasion. In other recent studies, however, similarity between the protagonist and the readership did influence identification and the persuasiveness of narratives [17, 18]. Even in almost identical experiments mixed results were found: Chen and colleagues [17] found significant, positive effects of a protagonist with the same age and of the same gender as their participants, while Chen and colleagues in 2017 [15] found no significant differences in a highly comparable experiment. The story topic was different (caffeine overdose in 2016 versus type 2 diabetes in 2017), but both studies manipulated age and gender of the protagonist, and used student participants.

A possible explanation for these contradictory results is that the manipulations of similarity may not have been perceived as such by the participants. Following Hoffner and Buchanan [19], who state that readers “tend to feel similar to characters who are like themselves in terms of demographic characteristics” (p. 328), many studies focus on the manipulation of such objective characteristics, like study program [18], gender and age [15, 17], or living conditions [20]. In general, these studies found significant but small effects of their similarity manipulation on perceived similarity. However, Cohen and colleagues [16] did not find significant effects of gender and nationality on perceived similarity in their first study, which they argue may have been because gender and nationality were not important to the general theme of the narrative used in their study. Likewise, it has been suggested that the effects of similarity are stronger when the similarity between a protagonist and receiver is on a story-relevant dimension rather than on a ‘simple’ demographic characteristic that is not important to the narrative [21]. Demographic similarities, such as age or gender, thus could increase identification but most probably so when they are relevant for the topic of the narrative ([18], but see also [16]).

### Current study

**Fig 1:**
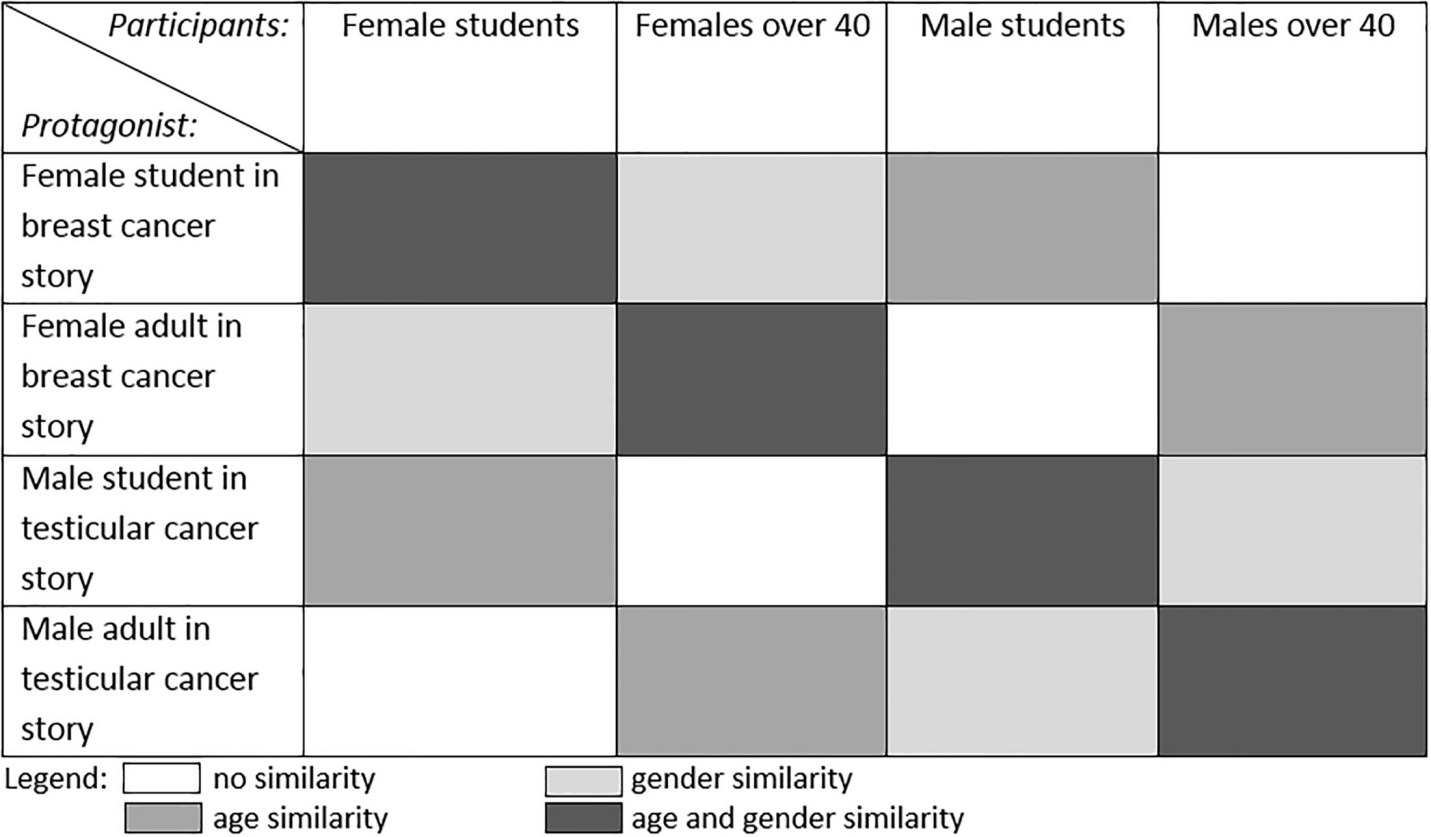
Overview of similarity conditions.

The current study aims to shed more light on the relationship between manipulated similarity and perceived similarity, and their effects on identification, transportation and persuasion. By persuasion, we mean the typical operationalization of changes in attitudes or behavioral intentions (cf. [7]). Similarity was manipulated by using two objective demographic characteristics—gender and age—that are crucially relevant for the story topics, which are testicular cancer (the most frequent form of cancer in young men), and breast cancer (the most frequent form of cancer in older women). Similarity in those demographics increases the perceived risk for the depicted health threat. In line with Chen and colleagues [15, 17], we thus focused on gender and age, but while they used these characteristics only *conjointly* (i.e. young male participants read the story version about a young male or an old female protagonist, and young female participants about a young female or an old male protagonist), we also investigated the *separate* effects of age and gender. More knowledge on these separate and possibly additive effects could help health communicators to decide how to tailor features of the protagonist of their message to the characteristics of the audience they want to reach. Therefore, we used four different combinations of these two objective characteristics. Fig 1 shows the four groups of manipulated similarity that result from these four combinations of story versions and participant groups that were used: 1) no similarity in age or gender, 2) only similarity in age, 3) only similarity in gender, and 4) similarity in both age and gender. With this design, we tested the following hypotheses, which we derived from the literature discussed above.

Hypothesis 1: A protagonist with the same age as the reader induces higher perceptions of similarity (1A), higher levels of identification (1B), higher levels of transportation (1C), and higher scores on persuasive measures (1D) than a protagonist with a different age.
Hypothesis 2: A protagonist with the same gender as the reader induces higher perceptions of similarity (2A), higher levels of identification (2B), higher levels of transportation (2C), and higher scores on persuasive measures (2D) than a protagonist with a different gender.

Cohen and Tal-Or [22] suggest that similarity manipulations may have additive persuasive effects. There is some evidence that the more characteristics are shared between the protagonist and the receiver of the narrative, the higher the narrative engagement [23], while there is no suggestion in the literature that leads us to expect interaction effects between age similarity and gender similarity. That is why we propose:

Hypothesis 3: Similarity in both age and gender leads to additive, and not interactive, effects on perceived similarity (3A), identification (3B), transportation (3C), and persuasive measures (3D).

Most previous studies into the effects of similarity were conducted with student participants [15–18, 20], but we were curious to see if results would be qualitatively different between adults and students. Additionally, earlier research found differential effects of narratives on males and females [7], making the question relevant whether results differ between men and women. Therefore, the role of age and gender of the participants is also examined in this study, but there was not enough evidence to formulate specific hypotheses concerning these participant characteristics.

Summarizing, to determine the effects of similarity on persuasive variables, we conducted an experiment in which participants read a narrative about a protagonist who was similar or dissimilar with the reader in age and/or gender. After reading, participants filled out a questionnaire with items measuring perceived similarity, identification, transportation, self-referencing, attitudes, and intentions, and also the emotions they felt while reading the narrative.

### Narrative processing

Besides examining the effects of similarity, we also investigated the process leading to the persuasiveness of narratives and the role of similarity therein. It has been suggested that transportation and identification impact attitudes and intentions through emotional responses that are evoked by the narrative [8, 9]. In an earlier study [24], we found that transportation and identification were indeed linked to emotions: transportation was positively associated with fear, sadness, surprise, and compassion, and identification was positively associated with compassion. In addition, we found that transportation was a significant predictor of behavioral intention. Of the emotions, only fear proved to significantly predict the intentions to follow the recommendation of performing a self-exam. See S1 Appendix for a graphic representation of these relationships as found in our prior study [24].

A variable that was not included in that earlier study [24], but that may also be relevant for explaining the processing of narratives is *self-referencing*. Self-referencing occurs when “information is processed by relating it to aspects of oneself” ([25]: p 17). A number of studies have found empirical evidence for effects of self-referencing. Self-referencing can mediate the effect of transportation on emotional responding [14, 26], and also the effect of transportation on intentions [26]. Additionally, self-referencing can mediate the relationship between identification and persuasiveness ([15], but see also [20]).

**Fig 2:**
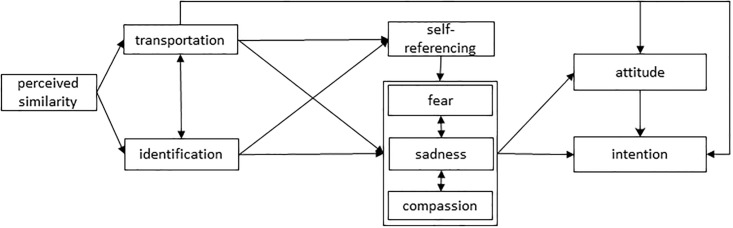
Hypothesized model of narrative persuasion.

In conclusion, it has been repeatedly shown that self-referencing contributes to narrative impact, but as this variable has so far not been included in theoretical models of narrative impact, its specific role in narrative processing remains rather unclear [27]. We decided to extend the model of narrative persuasion that resulted from previous work [24] and include self-referencing. This extended model, shown in Fig 2, was tested in this study.

## Method

### Materials

As health topic for the narratives we selected two types of cancer: testicular cancer (most frequent in young men), and breast cancer (most frequent in older women). We specifically focused on fear-arousing narratives in which we present a protagonist who suffers from cancer in an advanced state partly because he or she did not carry out the health check that was promoted by the message (cf. [28]). In the last paragraph of each version of the narrative, the protagonist urges the reader to perform a monthly self-exam to detect cancer at an early stage. Performing testicular exams can be useful for men of all ages, as testicular cancer is easily diagnosable by self-exams and 96% curable if it is detected in an early stage [29]. Most men currently do not perform self-exams [29] while self-exams are relatively easy, hence there is a need for persuasion. Performing breast self-exams is also the first step to cancer prevention, and is also an easy and no-cost tool that can be performed by women of all ages [30].

For the testicular cancer narrative, the protagonist was either a 22-year-old male student or a 50-year-old working father. For the breast cancer narrative, the protagonist was either a 22-year-old female student or a 50-year-old working mother. To reinforce the similarity manipulation, information on the lifestyle and living conditions of the protagonist was added, as can be seen in Table 1, showing the first paragraph of the narrative. All text versions were approximately 500 words long, in view of Cohen et al.’s warning [16] that in longer narratives the possibly rather subtle effects of similarity may be ‘eroded’ by effects of transportation. The narrative were presented as text only.

**Table 1:** Sample paragraphs of the four versions.

Female student protagonist	Female adult protagonist
*Breast cancer*	
My name is Anna and I am 22 years old. At this point in my life, I thought I had everything under control. I had a great boyfriend, a small but cozy room in the lively center of Groningen, studied law at the university, and many loyal friends. What I did not have under control was developing cancer. But not just any cancer, breast cancer to be specific. I knew that it is the most frequent form of cancer in older women, but I had no clue that young women can also get it, especially because I have no family history of breast cancer.	My name is Anna and I am 50 years old. At this point in my life, I thought I had everything under control. I had a great family, a beautiful detached house in a peaceful village called Aduard, a nice job at the office, and many loyal friends. What I did not have under control was developing cancer. But not just any cancer, breast cancer to be specific. I knew that this is the most frequent form of cancer in women of my age, but I had never expected that I would get it, especially because I have no family history of breast cancer.
Male student protagonist	Male adult protagonist
*Testicular cancer*	
My name is Jan and I am 22 years old. At this point in my life, I thought I had everything under control. I had a great girlfriend, a small but cozy room in the lively center of Groningen, studied law at the university, and many loyal friends. What I did not have under control was developing cancer. But not just any cancer, testicular cancer to be specific. I had no clue that it is the most frequent form of cancer in guys ages 15–30 and I never expected that I would get it, especially because I have no family history of testicular cancer.	My name is Jan and I am 50 years old. At this point in my life, I thought I had everything under control. I had a great family, a beautiful detached house in a peaceful village called Aduard, a nice job at the office, and many loyal friends. What I did not have under control was developing cancer. But not just any cancer, testicular cancer to be specific. I knew that it is the most frequent form of cancer in guys ages 15–30, but I never expected that someone from my age would get it, especially because I have no family history of testicular cancer.

*Note*. The underlined text indicates differences between the story versions

An English translation of the narrative about testicular cancer with the 22-year-old male protagonist can be found in S2 Appendix. The original Dutch materials and translated versions are available on request from the first author.

### Measures

Participants were asked to fill out the questionnaire immediately after reading one of the story versions. Unless noted otherwise, 7-point Likert scales were used, ranging from strongly disagree (1) to strongly agree (7). Please note that below, the approximate English translations are given of the original Dutch items in the order as presented in the questionnaire.

Intentions for two different behaviors were measured: *intention to perform self-exams* and *intention to donate to cancer charity*. Following Fishbein and Ajzen [31], both variables were measured with two items (“In the next half year I plan to / will perform the self-exam monthly / donate to cancer charity”). Cronbach’s alpha’s were.91, respectively.95. Obviously, only men were asked to respond to the questions regarding self-exams of the testicles and only women to the questions regarding self-exams of the breasts.

*Attitude towards performing self-exams* and *attitude towards donating to cancer charity* were measured by asking how “useful”, “good”, “important”, and “effective” participants found these behaviors [32]. Cronbach’s alpha’s were.90, respectively.91. In line with the intention measure, only men responded to questions regarding self-exams of the testicles and only women to questions regarding self-exams of the breasts. Consequently, the attitude and intention regarding performing self-exams were not subject to gender similarity manipulations.

As *fear*, *sadness*, and *compassion* seem specifically relevant in the context of cancer messages [24], these three emotions were measured by presenting the statement “While reading the story, I felt …”, followed by response scales ranging from 1 (not ‘emotion word’) to 7 (‘emotion word’). The emotion words for fear and sadness were derived from Dillard and colleagues [33]: “afraid”, “scared”, “worried” for fear (α = .92), and “sad”, “dreary”, “dismal” for sadness (α = .90). To measure compassion, we used the emotion words “pity”, “compassion” and “sympathy” (α = .84) [34].

*Perceived similarity* was measured with part of the ‘homophily scale’ of McCroskey et al. [35]. All four items of the *attitude* homophily scale (“X thinks like me” / “behaves like me” / “is similar to me” / “is like me”) were used. Three items (“X has social status like mine” / “has background similar to mine” / “has same lifestyle as me”) were added, based on the *background* homophily scale (α = .89).

*Identification* was measured with three items of Tal-Or and Cohen [36], namely “While reading the narrative, I could feel the emotions X portrayed”, “I think I have a good understanding of X”, and “I felt I could really get inside X’s head”. Furthermore, “I identified with X” [14] and “I imagined how it would be to be X” [37] were added. Cronbach’s alpha was.79.

*Transportation* was measured with the Transportation Scale—Short Form [38]. This scale consists of five items: “I could picture myself in the scene of the events described in the narrative”, “I was mentally involved in the narrative while reading it”, “I wanted to learn how the narrative ended”, “The narrative affected me emotionally”, and “While reading the narrative I had a vivid image of X” (α = .80).

*Self-referencing* was measured with four items: “The narrative reminded me to my own experiences”, “The narrative made me think about what could happen to me”, “During reading, I related the narrative to myself”, and “During reading, memories came up” [26]. Cronbach’s alpha was.76.

Finally, participants were asked some personal characteristics, such as their age, gender, and family history with cancer.

### Participants, design, and procedure

Two participant groups were targeted: students aged between 18 and 30, and adults older than 40 years. In total, 324 students from the University of Groningen participated. They filled out the questionnaire either in cubicles of a research lab or behind a PC during a course they took. Their mean age was 21.46 (*SD* = 2.28), ranging from 18 to 30 years, 42.6% male, 57.4% female. The older adults were recruited via the student participants and via an announcement on social media. In this way, 258 adults were reached. Their mean age was 56.83 (*SD* = 7.40), ranging from 40 to 78 years, 46.5% male, 53.5% female.

The experiment was conducted online using Qualtrics software. After having given written consent to participate, participants were randomly assigned to one of the four story versions (see Fig 1).

### Ethics approval

The study was approved by the Research Ethics Committee (CETO) of the Faculty of Arts, University of Groningen.

## Results

The following section is divided into two parts. First, the hypotheses concerning the effects of manipulated similarity are tested. Please note that, for brevity reasons, from here we will use the term *similarity* to indicate manipulated similarity. Otherwise, the term *perceived similarity* will be used. The second part focuses on testing the hypothesized model. A correlation matrix of all measured variables can be found in S3 Appendix.

### Effects of similarity

To test H1 and H2, analyses of variance (ANOVAs) were performed with age similarity (similar versus dissimilar), gender similarity (similar versus dissimilar), gender (male versus female) and age group of the participant (‘students’ of 18 to 30 years versus ‘adults’ above 40 years) as between-participants factors. Although there were no specific hypotheses concerning these latter two characteristics of participants, the influence of participants’ age and gender is examined by looking at interaction effects between age or gender similarity on the one hand and participant characteristics on the other. To mitigate the effects of an inflated Type I-error due to multiple testing, we used an alpha level of.01. An overview of mean scores and standard deviations for the similarity conditions by student and adult participants can be found in Tables 2 and 3. As the effects were not influenced by gender of the participants (see below), Tables 2 and 3 do not show mean scores for men and women separately. Table 4 summarizes the significant main effects and interaction effects we found. From this table, it can be deduced if similarity in age and similarity in gender have additive effects (H3), namely when effects for both age and gender similarity are significant and in the same direction. The combinations for which there were no significant effects for any of the variables were excluded from the table. For brevity reasons, *Age Similarity* is abbreviated to ‘AS’, *Gender Similarity* to ‘GS’, *Participants’ Age Group* to ‘A’, and *Participants’ Gender* to ‘G’. This section concludes with exploratory findings regarding the effects of similarity on self-referencing and emotions.

**Table 2:** Means and standard deviations for age similarity, measured on 7-point scale.

	Age Similarity			
	Students (*n* = 324)		Adults (*n* = 258)	
	Similar age group (*n* = 166)	Dissimilar age group (*n* = 158)	Similar age group (*n* = 125)	Dissimilar age group (*n* = 133)
Perceived similarity	4.16 (1.18)	3.05 (1.10)	2.98 (1.24)	3.02 (1.23)
Identification	4.91 (1.02)	4.48 (0.98)	4.04 (1.12)	4.55 (1.16)
Transportation	4.93 (0.98)	4.61 (1.16)	4.18 (1.16)	4.68 (1.09)
Attitude towards donation	5.81 (0.89)	5.63 (1.08)	5.58 (1.34)	5.72 (1.32)
Intention to donate	3.13 (1.32)	2.66 (1.43)	4.07 (2.05)	4.35 (1.92)
Attitude towards self-exams	6.48 (0.63)	6.44 (0.67)	6.27 (1.24)	6.54 (0.85)
Intention to self-exams	4.95 (1.28)	4.49 (1.59)	4.64 (1.83)	4.49 (1.90)
Self-referencing	3.93 (1.39)	3.63 (1.43)	3.57 (1.55)	3.77 (1.50)
Fear	3.98 (1.44)	3.69 (1.44)	3.07 (1.51)	3.24 (1.51)
Sadness	4.51 (1.48)	4.46 (1.37)	3.39 (1.66)	3.89 (1.64)
Compassion	5.79 (1.05)	5.61 (1.05)	4.80 (1.35)	5.50 (1.26)

**Table 3:** Means and standard deviations for gender similarity, measured on 7-point scale.

	Gender Similarity			
	Students (*n* = 324)		Adults (*n* = 258)	
	Similar gender (*n* = 165)	Dissimilar gender (*n* = 159)	Similar gender (*n* = 135)	Dissimilar gender (*n* = 123)
Perceived similarity	3.81 (1.27)	3.41 (1.22)	2.92 (1.14)	3.10 (1.32)
Identification	4.86 (0.98)	4.53 (1.04)	4.37 (1.11)	4.23 (1.22)
Transportation	4.95 (1.06)	4.58 (1.08)	4.59 (1.10)	4.27 (1.18)
Attitude towards donation	5.55 (1.03)	5.91 (0.92)	5.63 (1.29)	5.67 (1.38)
Intention to donate	2.68 (1.25)	3.13 (1.50)	4.39 (2.01)	4.03 (1.95)
Self-referencing	4.00 (1.32)	3.55 (1.48)	3.70 (1.52)	3.64 (1.54)
Fear	4.01 (1.41)	3.66 (1.47)	3.05 (1.47)	3.27 (1.55)
Sadness	4.36 (1.46)	4.62 (1.34)	3.47 (1.76)	3.85 (1.54)
Compassion	5.78 (1.09)	5.62 (1.01)	5.17 (1.38)	5.15 (1.31)

*Note*. The attitude and intention regarding performing self-exams were excluded for the gender similarity analysis.

**Table 4:** Summary of main and interaction effects.

	AS	GS	A	G	AS * GS	A * G	AS * A	GS * A	GS * G	AS * A * G	GS * A * G
Perceived similarity	X		X				X	X			
Identification			X				X				
Transportation		X	X				X		X		
Attitude towards donation		X		X							
Intention to donate			X	X			X				
Attitude towards self-exams				X		X					
Intention to self-exams						X				X	
Self-referencing									X		
Fear			X								
Sadness		X	X	X							
Compassion	X		X				X		X		

*Note*. X indicates a significant effect at.01 level. AS = age similarity, GS = gender similarity, A = participants’ age group, G = participants’ gender.

#### Age similarity

Our first hypothesis predicted that a protagonist with the same age as the reader induces higher perceptions of similarity (1A), higher levels of identification (1B) and transportation (1C), and higher scores on persuasive measures (1D) than a protagonist with a different age.

There was a significant interaction between Age Similarity and participants’ age group on perceived similarity (F(1,566) = 31.79, p < .001, partial η^2^ = .053): Student participants perceived themselves to be more similar to a same-age protagonist than to a different-age protagonist. Adult participants showed no differences in perceived similarity. We also found an interaction between Age Similarity with participants’ age group on identification (F(1,566) = 25.55, p < .001, partial η^2^ = .043) and transportation (F(1,566) = 18.39, p < .001, partial η^2^ = .031), where students had higher scores when protagonists were of the same age, and adults had higher scores when protagonists had a different age (i.e., were younger). Thus, part A, B and C of Hypothesis 1 only received support in case of students.

**Fig 3:**
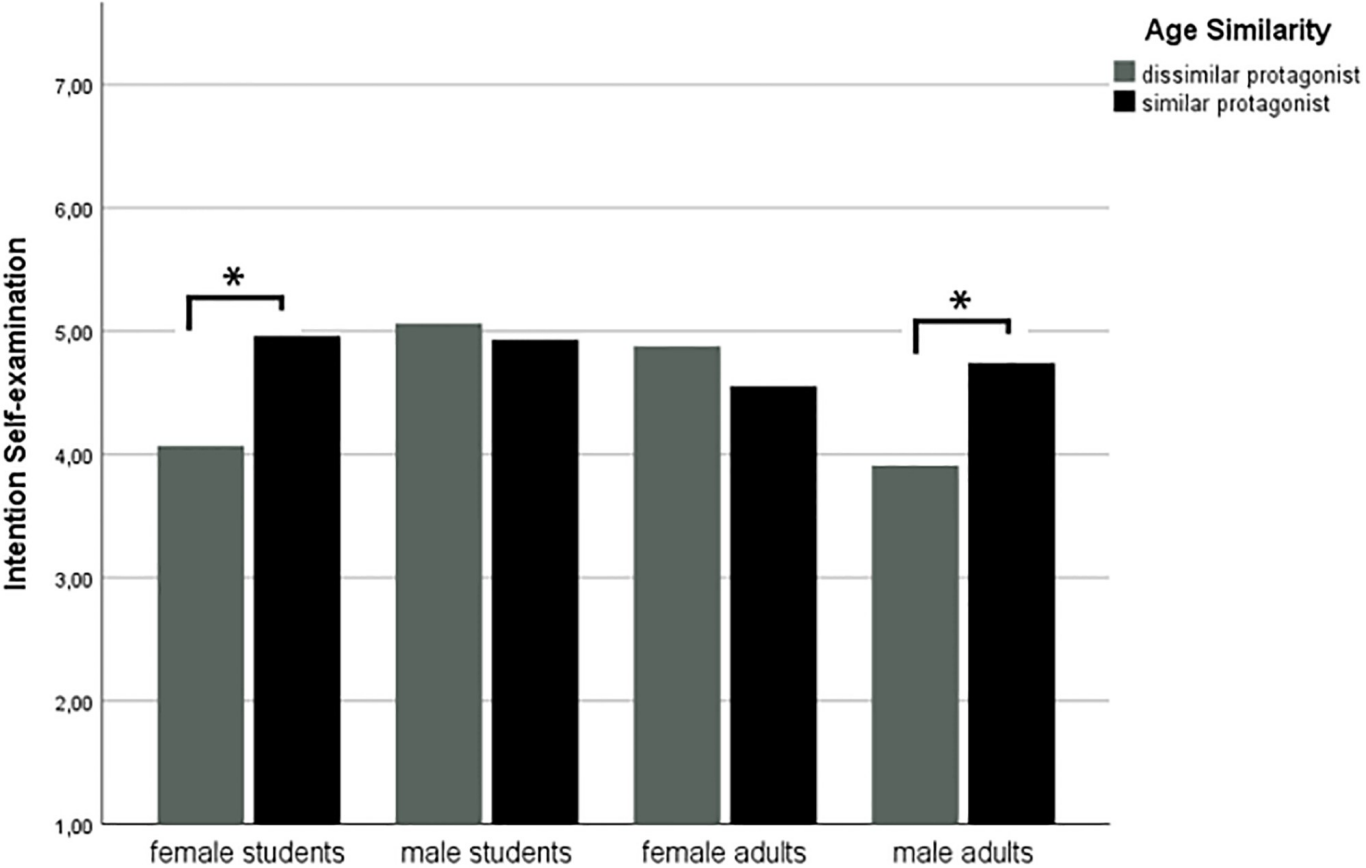
Bar graph of mean scores for intention to perform self-exams by age similarity, per participant group. *p < .01.

Part D of Hypothesis 1, concerning the persuasive measures, also only received partial support. There were no main effects of Age Similarity on attitude towards performing self-exams (p = .20) nor on attitude towards donating (p = .78), nor were there any interactions involving Age Similarity (p > .37). For donation intention, Age Similarity showed an interaction with participant’s age group (F(1, 566) = 7.49, p = .006, partial η^2^ = .013): for students, a same-age protagonist resulted in stronger intentions to donate than a different-age protagonist. For adults, no differences were found. In addition, there was a three-way interaction effect of Age Similarity, participants’ age group and participants’ gender on intention to perform self-exams (F(1,291) = 8.14, p = .005, partial η^2^ = .027). Fig 3 visualizes this three-way interaction by showing the mean scores per participant group. Female students (*M* = 4.96, *SD* = 1.29) and male adults (*M* = 4.74, *SD* = 1.83) were more inclined to perform self-exams after reading about a same-age protagonist than about a different-age protagonist (*M* = 4.07, *SD* = 1.64, respectively *M* = 3.91, *SD* = 2.04). For female adults and male students, difference in age of the protagonist did not lead to significant differences in intention.

#### Gender similarity

Our second hypothesis predicted that a protagonist with the same gender as the reader induces higher perceptions of similarity (2A), higher levels of identification (2B) and transportation (2C), and higher scores on persuasive measures (2D) than a protagonist with a different gender.

Gender Similarity only showed an interaction with participants’ age group on perceived similarity (F(1,566) = 6.32, p = .012, partial η^2^ = .011): Student participants perceived themselves to be more similar to a same-gender protagonist than to a different-gender protagonist, while adult participants again showed no differences in perceived similarity, partially supporting 2A. For identification and transportation, no main or interaction effects of Gender Similarity were found, providing no support for hypothesis 2B nor 2C.

Part D of hypothesis 2 was also not supported: There were no main effects of Gender Similarity on attitude towards donating or donation intention, nor were there interactions with participants’ age group or gender.

#### Additive effects

Our third hypothesis predicted that similarity in both age and gender lead to additive scores on perceived similarity (3A), identification (3B), transportation (3C), and persuasive measures (3D), as compared to similarity in only age or only gender. Just as in the previous analyses, we checked for the influence of the participant characteristics. There was one *additive* effect of Age Similarity and Gender Similarity, namely where perceived similarity was concerned: here, we found an interaction of Age Similarity with *Participants’ Age Group*, and an interaction of Gender Similarity with *Participants’ Age Group*, showing that, for student participants, a same-age and a same-gender protagonist led to more perceived similarity than a protagonist of different age or gender. Adult participants did show differences in perceived similarity.

#### Exploratory findings: Effects of similarity on self-referencing and emotions

Although there were no hypotheses concerning the effects of similarity on self-referencing and emotions, we did explore if self-referencing and the emotions of fear, sadness and compassion were influenced by the similarity manipulations. For self-referencing, there only was a significant interaction between Gender Similarity and participants’ gender (F(1,566) = 7.74, p = .006, partial η^2^ = .013). Only for women, a female protagonist led to more self-referencing than a male protagonist; for men, no significant differences were found. For fear, there were no significant main or interaction effects (p > .025).

For sadness, there was an interaction between Gender Similarity and participants’ gender (F(1,566) = 15.95, p < .001, partial η^2^ = .027). Men felt more sadness after reading about a different-gender protagonist than after reading about a same-gender protagonist. For women, no differences in sadness were found. For compassion, there was an interaction between Age Similarity and participants’ age (F(1,566) = 18.06, p < .001, partial η^2^ = .031), such that adults felt more compassion after reading about a different-age protagonist than a same-age protagonist. For students, no differences were found. Furthermore, there was a significant interaction between Gender Similarity and participants’ gender (F(1,566) = 6.84, p = .009, partial η^2^ = .012): Women reported more compassion after reading about a female protagonist than after reading about a male protagonist, while men reported equal levels of compassion.

### Role of perceived similarity in narrative processing: A SEM analysis

To examine the mechanisms underlying the processing of narratives, we used structural equation modeling with AMOS 25.0. We tested whether the model we hypothesized (see Fig 2) had adequate model fit according to the criteria of Kline [39]: (a) the model chi square divided by its degrees of freedom (*χ*^*2*^/*dƒ*) should be less than 3.0, (b) the comparative fit index (*CFI*) should exceed.90, and (c) the root mean square error of approximation (*RMSEA*) should be lower than.08. The model was first fitted to the data of the total sample, after which we checked for possible differences between the student sample and the adult sample by performing a multigroup analysis [24], as student and adult participants differed on nearly all variables that were measured (see Table 4). Please note that in the graphic representation of our models, covariance arrows are used to indicate the reciprocal relationships between transportation and identification, and between the emotions. In the path analyses, however, unidirectional relations were tested as required by AMOS.

**Fig 4:**
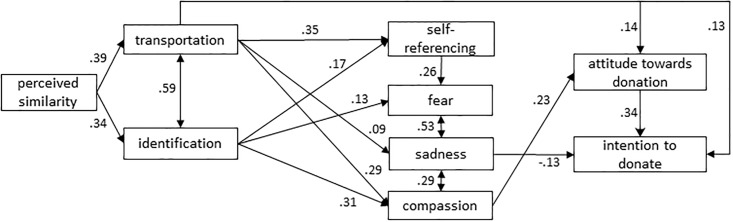
Final model concerning donation with standardized estimates (N = 582).

We started with the data of the total sample, with the attitude and intention concerning donation to cancer charity. The initial model had a *CFI* of.977 and an *RMSEA* of.073, but *χ*^*2*^/*dƒ* was with 4.12 not satisfactory. Following Byrne’s suggestion [40] to also evaluate the individual parameter estimates of paths, we deleted all non-significant paths (self-referencing to compassion and to sadness, transportation to fear, identification to sadness, fear to attitude towards donation, sadness to attitude towards donation, compassion to intention to donate, fear to intention to donate). This resulted in a good model fit: *χ*^*2*^/*dƒ* = 2.41, *CFI* = .985, *RMSEA* = .049. Fig 4 presents the final model concerning donation. The multigroup analysis did not provide evidence for differences in causal patterns for the two groups (i.e. student sample and adult sample), they only showed differences in *strength* for a number of relations. For students, the relationship between sadness and compassion (*β* = .48, *p* < .001) was stronger than for adults (*β* = .23, *p* < .001; *z* = 2.88, *p* < .01), while the relationship between fear and sadness was stronger for adults (*β* = .68, *p* < .001) in comparison to students (*β* = .40, *p* < .001; *z* = 4.31, *p* < .01). Furthermore, the estimate of the path from attitude to intention was significantly higher for adults (*β* = .64, *p* < .001) than for students (*β* = .35, *p* < .001; *z* = 2.56, *p* < .05). No other significant differences were found, indicating that the paths in the model were consistent between the two samples.

**Fig 5:**
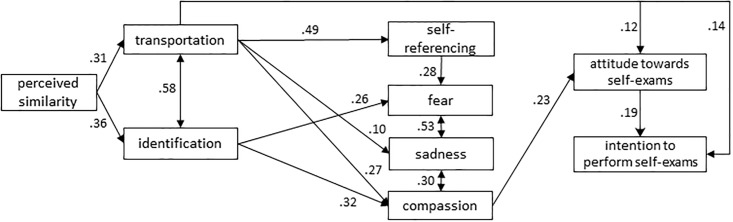
Final model concerning performing self-exams with standardized estimates (n = 299).

Next, we evaluated the fit of that same model in Fig 4 with the data for attitude and intention towards performing self-exams as outcome variables (*n* = 299, as men only answered questions on testicular self-exams, and only women the questions on breast self-exams). Model fit turned out to be good: *χ*^*2*^/*dƒ* = 1.67, *CFI* = .985, *RMSEA* = .047. Two paths (identification to self-referencing, and sadness to intention) were non-significant and were hence deleted. Fig 5 presents the final model concerning performing self-exams (*χ*^*2*^/*dƒ* = 1.71, *CFI* = .982, *RMSEA* = .049). The multigroup analysis again showed that the relationship between sadness and compassion was stronger for students (*β* = .55, *p* < .001) than for adults (*β* = .24, *p* < .01; *z* = 2.63, *p* < .01), while the relationship between fear and sadness was stronger for adults (*β* = .72, *p* < .001) in comparison to students (*β* = .45, *p* < .001; *z* = 2.89, *p* < .01). In addition, we found that the path from transportation to sadness was significant for adults (*β* = .31, *p* < .01), but not for students (*β* = .01, *p* = .91; *z* = 2.18, *p* < .05). Finally, the path from attitude to intention was significant for students (*β* = .75, *p* < .001), while it was only marginally significant for adults (*β* = .28, *p* = .06; *z* = -2.14, *p* < .05). No other significant differences were found, indicating that most paths in the model were consistent between the two samples.

Both models show that, in conformity with the hypothesized model in Fig 2, perceived similarity was positively associated with transportation and identification. The three emotions we measured proved to be predicted by transportation or identification, and fear also by self-referencing. Of these emotions, only compassion predicted attitudes; sadness influenced only the intention to donate. Transportation turned out to be a significant predictor of attitudes and intentions towards both donation and performing self-exams.

## Discussion

In this study, we investigated whether manipulated similarity with a protagonist affected the persuasive outcomes of narratives, through perceived similarity, transportation, identification, self-referencing, and the emotions of fear, compassion, and sadness. We conducted an experiment in which we varied the age and gender of the story protagonist to make him/her less or more similar to the participants, who were females and males, either of student age or above 40 years old (further abbreviated to ‘students’ versus ‘adults’). Similarity in age and similarity in gender were assumed to be relevant to the chosen health theme of cancer, since the susceptibility for breast cancer and testicular cancer is age and gender sensitive: breast cancer most often affects older women, and testicular cancer is most often found in young men. We hypothesized that a protagonist with the same age and gender as the reader would induce higher perceptions of similarity, higher levels of identification and transportation, and higher scores on persuasive measures than a protagonist with a different age and gender.

### Age similarity

In line with our first hypothesis, we found that age similarity produced differences in perceived similarity, but only for student participants. As expected, student participants felt more similar to a student protagonist instead of an adult protagonist. For adult participants, there were no significant differences in perceived similarity.

With regard to the narrative variables we measured, age similarity again appeared to be especially relevant for students, who felt more transported and could identify more with a student than with an adult protagonist. These findings are not surprising, because peers play an important role in the lives of young people. More surprising perhaps was the finding that also adults reported more identification, transportation, and compassion after reading about a student protagonist than after reading about a protagonist of their own age. Possibly, some adults were more touched by the fate of a young person than that of a protagonist of their own age, because some of them might have children of the same age as the young protagonists (i.e., around 20 years of age) or because of the general conviction that especially young people should not get serious diseases such as cancer. Another possible explication may lie in ‘age denial’: people aged over 40 often perceive themselves as younger than they truly are [41], causing some adults to connect with a younger protagonist.

Similarity in age led to persuasive effects for specific groups of participants. Student participants were more inclined to donate after they read about a protagonist of similar age, while adults were willing to donate regardless of the age of the protagonist. Regarding intention to perform self-exams, age similarity positively affected only female students and male adults. Perhaps reading about someone of their own age made participants in these two groups suddenly realize that they were also at risk for getting cancer, precisely because it is normally older females who get breast cancer, and not young female students, and it is generally younger males who are prone to testicular cancer, and not older men.

### Gender similarity and additive effects

Hardly any effects were found for gender similarity, providing very little support for hypothesis 2. Gender similarity had no effect on persuasive measures. Gender similarity only produced differences in perceived similarity for student participants. Consistent with age similarity, student participants felt more similar to a protagonist of similar gender, while adult participants did not show differences.

Besides separate effects of age and gender similarity, we expected that the condition with similarity in both age and gender would lead to additive scores on persuasive measures (H3), especially since age as well as gender were relevant characteristics for the narrative at hand. However, having both age and gender in common with the protagonist did not lead to higher scores on attitudes or intentions than sharing only one of these characteristics with the protagonist. Only perceived similarity was affected by both age and gender similarity.

### Processing of narratives: Evaluating the SEM model

In the present study, we also examined the fit of a model we developed for the processing of narratives, largely based on the model in an earlier study [24]. We extended that model to include two new concepts: perceived similarity and self-referencing. Path analyses largely supported the proposed model across the student sample as well as the adult sample: Perceived similarity positively predicted both transportation and identification, which in turn predicted fear, sadness and compassion. Transportation also predicted attitudes and intentions directly, suggesting that transportation, and not identification, is central to narrative persuasion (for similar findings, see [11, 24]). Transportation, and in one of the models also identification, also positively predicted self-referencing. Self-referencing, however, did not predict persuasive outcomes, neither directly nor via emotions. Self-referencing was only related to the emotion of fear, and in our current model, fear was not a direct predictor of behavioral intentions. This latter finding contrasts with our earlier model [24], in which fear did increase intention to perform self-exams (see S1 Appendix). Interestingly, in the present study, compassion seems to have taken over the persuasive power of fear, as this time, compassion did influence behavioral intentions (indirectly via attitudes). With regard to donation behavior, this may be explained by the so-called ‘action tendency’ of compassion [42]. Compassion typically leads to the desire to help others, for example, by donating money to charity. It is much less clear, however, why compassion was also beneficial for a self-focused behavior such as performing self-exams. More research is needed in this area. Future research might also further examine the role of sadness. We found that the path from transportation to sadness was weak and did not even exist for students in one of the models. Additionally, we found that the emotion of sadness directly influenced intention, but only for the intention to donate and with a small, negative coefficient. The lack of persuasive effects for sadness could be explained by the action tendency of sadness, which is withdrawal [33], suggesting that sad people do not take action.

### Limitations

Some limitations must be acknowledged, especially with regard to the kind of narrative that was used in this study. Our fear-arousing narrative resembles narratives used in public health campaigns, but these narratives might be different from narratives that people naturally share, nowadays increasingly via social media. Despite the possible differences, research has shown that both stories that come from traditional media, such as health campaigns, as well as stories from social media often focus on the harms of diseases, just like the narratives in our study did [43]. To increase the external validity of our results, more research is needed. Another possible limitation is that though there were four story versions, there was only one particular storyline. This obviously can limit the generalizability of our research findings, and it can be especially problematic for anecdotal experiences such as narratives, which then portray only one experience on which readers can base their health decisions [44]. To properly guide individuals through their health decision making process, Holmberg [44] advises to present storylines that take both the subjective experiences of patients and medical knowledge into account. Our narrative indeed contained doctor’s advice, which may be regarded as medical knowledge. As a final limitation of this study, it must be noted that the effect sizes, reported as partial eta squared, did not exceed the benchmark of.0588 that Cohen [45] proposes for medium effects. This implies that the reported differences only represent small effects. Even small effects of mass media, however, suggest that the health behavior of large numbers of people could be affected [6].

### Implications

Our outcomes provide several important insights with regard to the effects of similarity manipulations and the processing of narratives. First, this study shows that, for student participants, the age characteristic was more powerful than gender. This advantage of age similarity over gender similarity could explain the mixed results of Chen and colleagues [15, 17], who combined age and gender in their similarity manipulation. They only found positive effects for a narrative on caffeine overdose which “poses proximal effects for young audiences” ([15]: p 911), making age similarity more relevant than gender similarity. In the other study, the topic was type 2 diabetes, which can afflict people of any age, but especially older people. For future studies, it is recommended to disentangle age and gender in order to make clear which characteristic caused which effects. Second, we discovered that student and adult participants differed on nearly all variables that were measured. With only students as participants, as in many earlier studies [15–18, 20], we would likely have assumed, incorrectly, that age similarity is of importance for any audience. An important strength of the present study is thus that also adults participated. For these adults, having the same age as the protagonist they read about did not matter too much. Sometimes, adults even had a more positive reaction to a young protagonist than to a same-aged one. Thus, our findings suggest that researchers should be very careful with generalizing from findings gathered from student samples to the general population. These findings also have practical implications. As has become apparent from our results, health campaign designers should definitely take the age of the audience into account when designing health messages. However, in cases where tailoring is impractical or impossible [46], campaigners may want to choose for young protagonists, because having a young main character was most persuasive for students and did not negatively affect the persuasive results for older people.

In spite of the different effects of the similarity manipulations on student and adult participants, we found that students and adults process narratives in a similar way. The findings of the path analyses suggest that perceived similarity should be added to models of narrative persuasion. For the concept of self-referencing, no such support was found. In line with earlier research [27], we found that perceived similarity can positively predict narrative persuasion, as perceived similarity was related to both transportation and identification, which, in turn, were related to attitudes and health behavioral intentions, directly—in the case of transportation—or indirectly, via the emotion of compassion.

### Conclusion

In conclusion, even though earlier research [2, 15, 16] casted doubt on the effectiveness of similarity manipulations, our study shows that both manipulated and perceived similarity are important factors in narrative persuasion. We found that manipulated similarity can expand the persuasive power of a narrative, be it for specific participant groups and similarity manipulations: Particularly age similarity was found to be important for young people. Further research is needed to generalize this finding beyond the specific context of this study. For all participants, perceived similarity influenced the mechanisms underlying narrative persuasion. Thus, similarity should be kept on the research agenda of health communication.

## Supporting information

S1 Appendix Final model in Authors (XXXX). (TIF)

S2 Appendix Narrative with male student protagonist. (DOCX)

S3 Appendix Correlation matrix (*N* = 582). (DOCX)
