# Supplementary material for: “Hey, that could be me”: The role of similarity in narrative persuasion
Source: PLoS One. 2019 Apr 18;14(4):e0215359. doi: 10.1371/journal.pone.0215359 (PMC6472763; doi:10.1371/journal.pone.0215359)
Supplement: S2 Appendix — (DOCX) [file pone.0215359.s002.docx]

**S2 Appendix. Narrative with male student protagonist.**

My name is Jan and I am 22 years old. At this point in my life, I thought I had everything under control. I had a great girlfriend, a small but cozy room in the lively center of Groningen, studied law at the University of Groningen, and had many loyal friends. What I didn’t have under control was developing cancer. But not just any cancer, testicular cancer to be specific. I had no clue that it is the most common form of cancer in guys ages 15–30 and I never expected that I would get it, especially because I have no family of testicular cancer. I started having some pain in my left testicle. After about a month, I finally went to the doctor because my mother pushed me to. To my horror, the doctor found cancer.

***Metastasized***

Only one week after the diagnosis, my left testicle was removed. But that didn’t stop the misery. My relationship was getting worse. We didn’t talk to each other and we could no longer have sex because I had trouble getting and keeping an erection. With only one testicle, I felt so embarrassed. We broke up last week. I dropped out of my college, fraternity, and soccer team, mainly because I didn’t have any energy left and because my so-called friends started treating me different. The chemotherapy made me lose my hair, and I feel sick all the time. But that’s not all. The really bad news is that the cancer has spread to my liver, and the doctor gives me only a small chance of beating it. I keep thinking, I am only 22 years old, this isn’t supposed to be happening!

***Too Macho***

If I had discovered the tumor earlier, I might have really improved my chances of beating cancer. I had heard of the testicular self-exam (TSE) to check for tumors, but it seemed nonsense to me. None of my friends ever talked about it, and I guess none of them did the exam either. Now, what do I have to show for my macho attitude? No girlfriend, no sex, no hair, and barely any friends. And I’m terminally ill. Perhaps, I won’t survive.

***Better Early Than (Too) Late***

Dear reader, don’t make my mistake. Don’t let your pride and ego keep you from doing the self-exam. It’s really simple: Once a month, after a warm shower, roll each testicle gently between your thumb and fingers. Feel for any hard lumps. If you have pain in your testicles, or if you find a lump, go see your doctor immediately. He tells me there is a big chance of survival if it is caught early.

Learn the TSE and do it once a month. It will save your life!
