# Supplementary material for: “Hey, that could be me”: The role of similarity in narrative persuasion
Source: PLoS One. 2019 Apr 18;14(4):e0215359. doi: 10.1371/journal.pone.0215359 (PMC6472763; doi:10.1371/journal.pone.0215359)
Supplement: S3 Appendix — (DOCX) [file pone.0215359.s003.docx]

**S3 Appendix. Correlation matrix (*N* = 582).**

|  | PS | Id | Tr | SR | Fe | Sa | Co | ADo | IDo | ASE | ISE |
| --- | --- | --- | --- | --- | --- | --- | --- | --- | --- | --- | --- |
| Perceived similarity (PS) | - | .57** | .39** | .28** | .33** | .23** | .27** | .13** | -.06 | .09 | .07 |
| Identification (Id) |  | - | .72** | .42** | .39** | .39** | .52** | .21** | .09* | .20** | .23** |
| Transportation (Tr) |  |  | - | .47** | .37** | .42** | .52** | .26** | .16** | .23** | .27** |
| Self-referencing (SR) |  |  |  | - | .38** | .31** | .24** | .13** | .11* | .09 | .24** |
| Fear (Fe) |  |  |  |  | - | .63** | .29** | .11** | .01 | .09 | .16** |
| Sadness (Sa) |  |  |  |  |  | - | .48** | .13** | -.04 | .16** | .23** |
| Compassion (Co) |  |  |  |  |  |  | - | .30** | .12** | .29** | .30** |
| Attitude donation (ADo) |  |  |  |  |  |  |  | - | .35** | .30** | .05 |
| Intention donation (IDo) |  |  |  |  |  |  |  |  | - | -.00 | .18** |
| Attitude Self-exams (ASE)^$^ |  |  |  |  |  |  |  |  |  | - | .27** |
| Intention Self-exams (ISE) ^$^ |  |  |  |  |  |  |  |  |  |  | - |

Note. **p* < .05, ***p* < .01; ^$^(*N*=299)
